# The influence of ripening on the nutrient composition and antioxidant properties of New Zealand damson plums

**DOI:** 10.1002/fsn3.4097

**Published:** 2024-03-30

**Authors:** Ali Rashidinejad, Mirja Kaizer Ahmmed

**Affiliations:** ^1^ Riddet Institute Massey University Palmerston North New Zealand; ^2^ Present address: Faculty of Fisheries Chattogram Veterinary and Animal Sciences University Chattogram Bangladesh

**Keywords:** antioxidants, bioactive compounds, harvesting effect, New Zealand edible plums, nutrients, proximate composition

## Abstract

The current study pioneers a comprehensive exploration into the influence of ripening stages on the nutritional composition and antioxidant attributes of the New Zealand damson plums (*Prunus domestica ssp. Insititia*). Sampled at early‐, mid‐, and late‐ripening stages from randomly selected plum trees, the investigation unveiled notable significant (*p* < .05) declines in multiple parameters as ripening progressed. Noteworthy reductions in dry matter (from 21% to 19.33%), stone weight (from 30.23% to 24.30%), total dietary fiber (from 3.15% to 2.5%), energy content (from 280 to 263 kJ/100 g), vitamin D3 (from 1.67 to 1.53 μg/100 g), vitamin A (from 4.2 to 3.87 μg/100 g), and specific minerals (e.g., Ca, Mg, and P) emerged as a hallmark of this progression. Additionally, plums harvested at the advanced ripening stage exhibited heightened moisture content in contrast to their early‐stage counterparts. Conversely, ash, protein, carbohydrates, total sugar, and minerals (including K, Na, Zn, and Se) demonstrated no significant alteration (*p* > .05) across ripening stages. Remarkably, damson plums that were harvested at the end of the ripening stage displayed reduced phenolic content and total antioxidant activity compared to those acquired at the early–mid ripening phase. This research collectively highlights the substantive impact of harvesting time and ripening stage on the nutritional and antioxidant profiles of damson plums cultivated in New Zealand.

## Graphical abstract

Total antioxidant activity (TAA; A) and total phenolic content (TPC; B) of fresh damson plums at different stages of ripening.

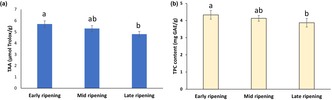

## 1 INTRODUCTION

Purple fruits, including blackcurrants, blueberries, and plums, are well‐known for being rich sources of several bioactive compounds, such as anthocyanins, vitamins (A, C, and E), phenolic compounds, and carotenoids. These compounds are reported to exhibit numerous health benefits, including antioxidant, anti‐inflammatory, and anticancer properties (Cortez & Gonzalez de Mejia, 2019; Costa et al., 2013; Karaman et al., 2013; Khoo et al., 2019). The anthocyanins from these types of fruits help generate retinal pigments, improve blood circulation within the retinal capillaries, and reduce the risk of macular degeneration (Khoo et al., 2019).

Damson plums, scientifically known as *Prunus domestica* ssp. Insititia, are a subspecies of *Prunus domestica*. Historically referred to as ‘damascene,’ a nod to their Syrian origin, these relatively small, ovoid‐shaped fruits measure about 20–25 mm in size. These are characterized by a distinctive and somewhat astringent taste, making them a popular choice for culinary applications, especially in the creation of fruit preserves and jams (Schmidt‐Tauscher et al., 1996). Damson plums are distinguished by their unique stone, both in morphology and genetics, which sets them apart from other plum varieties. These plums, which are known for their deep purple hue, are cultivated worldwide, with a significant presence in regions such as Europe (e.g., Italy, Greece, and Turkey), western Asia, southern Russia, North Africa, and Anatolia (Woldring, 2000). In New Zealand, damson plums are cultivated particularly in the region of Hawke's Bay. As a purple fruit, damson plums are likely to contain elevated concentrations of bioactive compounds, including anthocyanins. Such compounds hold potential for use in functional foods or as a functional ingredient in nutracutical products. Additionally, damson plums can serve as a valuable source for the extraction and isolation of specific bioactive compounds.

Nonetheless, it is crucial to highlight that the prevailing literature so far has primarily focused on scrutinizing organic acids, soluble sugars, phenolic compounds, and specific genetic traits inherent to damson plums. Yet, a deeper investigation into their bioactive constituents and potential applications remains an uncharted realm demanding exploration (Fernandez‐Otero et al., 2006; Fernández‐Otero et al., 2007; Garcia‐Marino et al., 2008; Iglesias‐Fernández et al., 2007; Mousavi et al., 2019). Remarkably, there has been a notable absence of systematic publications or reports regarding the nutritional or antioxidant properties of damson plums up to the present. Furthermore, the influence of various ripening stages on these properties remains unexplored.

Recently (Xia et al., 2023), for the first time, we demonstrated that the New Zealand damson plums are a valuable source of bioactive compounds with potent antioxidant properties. The study also evaluated the impact of different solvents and extraction methods on the phenolic compounds present in these plums. Therefore, the present study was designed to comprehensively investigate the nutritional composition, including proximate composition, sugar profiles, vitamins, and minerals, as well as the antioxidant properties of damson plums across their early‐, mid‐, and late‐ripening stages. This study aimed to explore the impact of various ripening stages on the nutritional value and antioxidant properties of damson plums. Our research hypothesis that significant variations will be observed in the nutritional composition, antioxidant capabilities, and concentration of bioactive compounds in damson plums from New Zealand as they progress through different stages of ripening.

## 2 MATERIALS AND METHODS

### 2.1 Chemicals

Methanol, 6‐hydroxy‐2,5,8‐tetramethylchroman‐2‐carboxylic acid (Trolox), Folin–Ciocâlteu reagent, gallic acid, and ABTS (2,2′‐azino‐bis(3‐ethylbenzothiazoline‐6‐sulfonic acid) were purchased from Sigma‐Aldrich (St Louis, MO, USA). Deionized water was obtained from a Milli‐Q Ultra‐pure water system (Millipore, Billerica, MA, USA). All chemicals and reagents were of analytical grade and were used as received without any further purification.

### 2.2 Sample preparation

**FIGURE 1:**
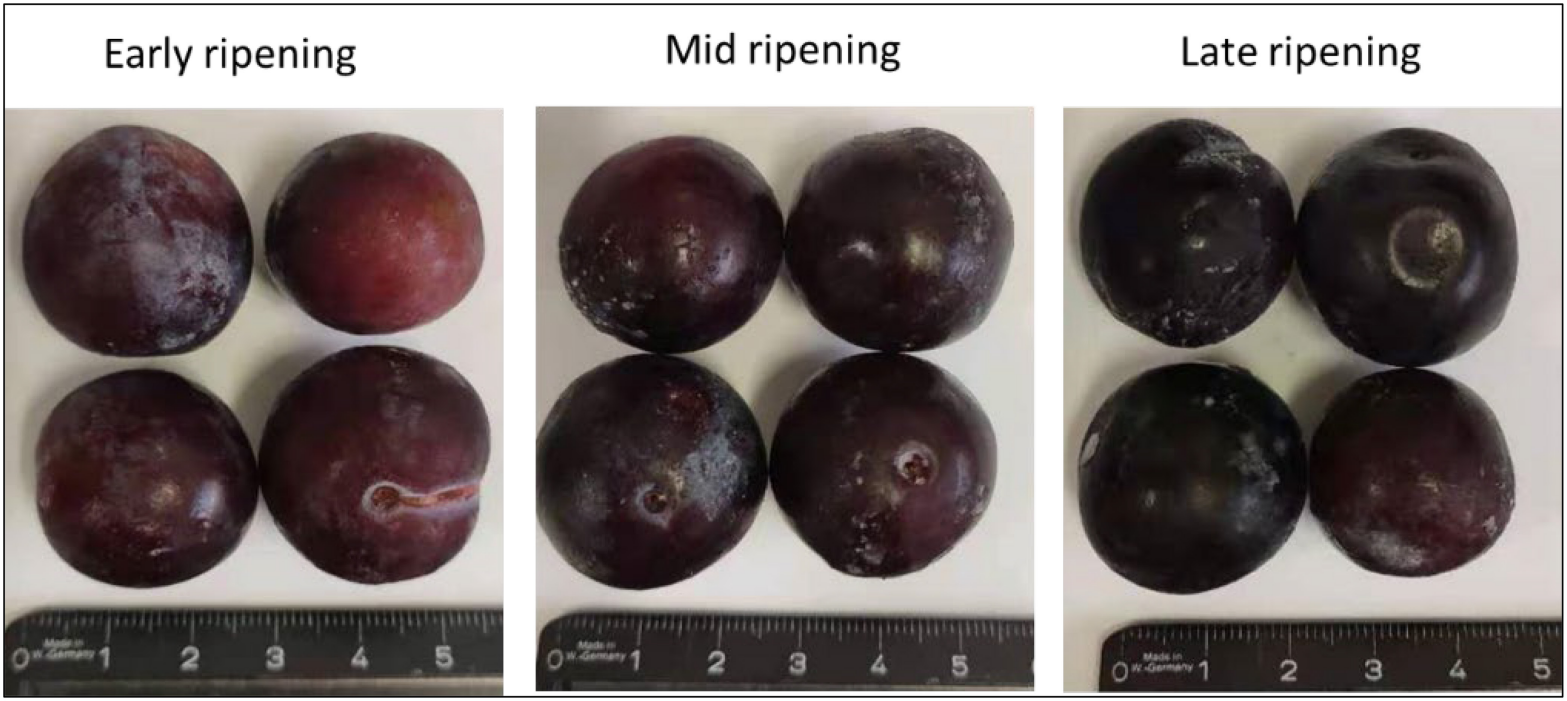
Visual representations of damson plums (from the same tree) at early‐, mid‐, and late‐ripening stages.

The fresh damson plums (*Prunus domestica*, ssp. Insititia) were harvested by hand from an orchard belonging to Foot Steps Limited in Karamu, Hastings (39.6467° S, 176.8663° E), New Zealand. Trees (>5 years old and >3 meters apart) were selected randomly for sampling, with three kilograms of fresh plums collected in triplicates from each tree throughout the project following the complete randomization of all trees in the orchard. Samples representing early‐, mid‐, and late‐ripening stages were collected on the 2nd of February 2021, 11th of February 2021, and 24th of February 2021, respectively. Figure 1 visually illustrates the fruit samples harvested at three distinct ripening stages of damson plums. After the harvest, the plums were transferred (3–5 h transport time) to the laboratory in sealed plastic bags at room temperature. Upon arrival, the samples were stored in a freezer (−18°C), and subsequently, they were freeze‐dried using a Cuddon FD18 Freeze Drier (Cuddon Freeze Dry in Blenheim, New Zealand).

The pH of the fresh plums was measured using a pH meter (H1221, Hanna Instruments Ltd., Bedfordshire). This measurement was conducted by seperating the flesh, removing the stone, and creating a fruit paste. After removing the stone and freeze drying, the flesh was ground and passed through a 50‐mesh sieve to achieve a finely homogeneous powder. This powder was then packed in airtight containers and stored at −20°C until the corresponding analyses. The weight ratio of the stone to the flesh was calculated using the following equation:

(1) $$\text{Stone}/\text{flesh ratio}\;\left(\%\right)=\frac{\mathrm{Dry}\;\text{weight of stones}\;}{\mathrm{Dry}\;\text{weight of plums}}\times 100$$

### 2.3 Determination of proximate composition, fiber, and energy

The proximate compositions of damson plums were analyzed according to the standard AOAC methods (AOAC, 1990). Various established methods were employed for the comprehensive analysis of nutritional components in the damson plums. Protein and fat content were determined following AOAC methods 968.06 and 922.06, respectively, ensuring precise quantification of these essential macronutrients. Moisture content was assessed through gravimetric determination using the oven‐drying method, adhering to AOAC methods 925.10 and 930.15, guaranteeing accurate measurement of water content. Mineral composition, indicated by ash content, was determined after subjecting the samples to 6 h of heating at 550°C, aligning with AOAC method 942.05. Carbohydrate content was calculated by deducting the summation of lipid, protein, moisture, and ash contents from the total (100%), following AOAC method 2017.16, allowing for precise carbohydrate assessment. Fiber classification was carried out per the standard AOAC method 991.43 with adherence to Megazyme guidelines, ensuring accurate classification of dietary fiber components. Additionally, energy content was calculated based on the macro‐nutrient data, providing a comprehensive understanding of the overall nutritional value of the damson plums. These rigorous and standardized methodologies guarantee the accuracy and reliability of the nutritional analyses conducted on these fruits.

### 2.4 Determination of sugar profiles

The sugar profile was determined using gas chromatography with the Flame Ionization Detector (GC‐FID), according to the method described previously (Nadeem et al., 2019). In the analytical process, 0.6 mg of the samples underwent incubation at 70°C for 5 min within a water bath containing hydroxylamine hydrochloride (12.5 mg) and pyridine (0.5 mL, 99% purity; Sigma‐Aldrich). Subsequently, the resulting oximes were converted into trimethylsilyl (TMS) derivatives by introducing hexamethyldisilazane (0.5 mL, 99% purity, Sigma‐Aldrich, Auckland, New Zealand) and trifluoroacetic acid (0.4 mL, 98% purity, Sigma‐Aldrich). To facilitate phase separation, isooctane (1 mL, 99% purity; Sigma‐Aldrich, Auckland, New Zealand) and deionized water were introduced into the test tube. This resulted in the dissolution of derivatized sugars in the upper organic layer. Subsequently, the isooctane layer, containing the volatile derivatives, was carefully transferred into a GC vial for subsequent analysis. To ensure accuracy, the volatile derivatives were analyzed relative to an internal standard (β‐d‐glucopyranoside, >98% purity; Sigma‐Aldrich, Auckland, New Zealand), which had been incorporated into the sample within the test tube prior to derivatization.

### 2.5 Vitamin and mineral profiles

Vitamin and mineral analyses were carried out in a certified lab located at Massey University, Palmerston North, New Zealand. Vitamins A and E were determined according to the modified AOAC method (974.29, 2001.13, Modified). Vitamin C was determined using high‐performance liquid chromatography (HPLC), according to the method described earlier (Lee & Coates, 1987). The method reported by Hines et al. (1986) was used for the quantification of vitamin D3.

### 2.6 Total phenolic content

Total phenolic content was determined according to the original Folin–Ciocâlteu assay (Singleton et al., 1999), adapted for microplate readers (BioTek, Winooski, VT, USA) (Rashidinejad et al., 2013). An aliquot of 20 μL of the sample extracts was transferred into a 96‐well microplate and 100 μL of Folin–Ciocâlteu reagent (0.2 N) was added. The mixture was allowed to react for 5 min before adding 80 μL of Na_2_CO_3_ solution (7%, w/v). The reaction mixture was incubated at 25°C in a dark place for 30 min, and the optical density was measured at 760 nm and calculated against a standard prepared with different concentrations (0–1000 μg/mL) of gallic acid. The results are expressed as mg gallic acid equivalents (GAE) per gram of dry plum sample.

### 2.7 Total antioxidant activity (TAA) evaluation

The freeze‐dried plum powder (8 g) was homogenized in 40 mL of water: ethanol (20:80, v/v) mixture at 250 rpm using a high‐shear mixer (Ultratrax T20, IKA‐Werke GmbH and Co. KG, Stufen, Germany). Then, the sample was centrifuged at 1900 *× g* for 10 min to collect the supernatant, which was used for the ABTS (2,2′‐azino‐bis(3‐ethylbenzothiazoline‐6‐sulfonic acid)) antioxidant assay using an ABTS Antioxidant Kit (100T/96S, Ruifan Biological Technology Company Ltd., China). The ABTS assay is recommended as one of the most accurate methods for the determination of TAA of plant samples (Gülçin, 2012).

The antioxidant kit came with a specific recipe for three different reagents (Reagent A, Reagent B, and Reagent C). To begin the assay, 11 mL of Reagent A was added to one bottle of Reagent B to form a radical cation (ABTS+) solution. This working solution was vortexed for 20 min to ensure complete mixing. The ABTS+ working solution was made fresh before every use. For sample preparation, 1 g of a dry sample of damson plums was used for the extraction of antioxidants using 1 mL of Reagent C. The mixture was then centrifuged for 10 min at 10,000 *g*, and the supernatant was transferred into a 96‐well microplate for the corresponding analysis. A 10 μL of an aliquot of sample solutions was taken into microplate wells, to which 190 μL of ABTS+ working solution was added. After 5 min of incubation in the dark (room temperature), the values of absorbance were recorded using a microplate reader (Multiscan GO; Thermo Scientific, Waltham, MA, USA) at *λ* = 734 nm. Mean values from three independent samples were determined for each extract. The TAA was calculated according to the following equation (Equation 2).

(2) $$\begin{array}{c} \text{Total antioxidant activity}\;\left(\text{μmol Trolox}/g\right)\\ =\frac{\left(\text{Blank absorbance}-\text{Sample absorbance}+0.0012\right)}{\text{Sample weight}\;\left(g\right)}\times 1.424 \end{array}$$

where, 0.0012 is adjustment factor and 1.424 denotes the calibration factor.

### 2.8 Statistical analysis

All tests were performed at least in triplicate. Data are expressed as mean ± standard deviation. One‐way analysis of variance (ANOVA) and Tukey's multiple comparisons test were performed to analyze the experimental data using the GraphPad Prism software (Version 6.01, San Diego, CA, USA). Differences were considered statistically significant at *p* <.05.

## 3 RESULTS AND DISCUSSIONS

In this study, a comprehensive analysis of proximate composition (moisture, ash, fat, protein, carbohydrates) and a wide range of nutrients, sugar profiles, total dietary fiber (TDF), insoluble dietary fiber (IDF), soluble dietary fiber (SDF), minerals, vitamins, and energy content in New Zealand‐grown damson plums was conducted. Furthermore, the antioxidant properties of the plum samples were investigated. The resulting nutritional profile was then compared across three different ripening stages to gain insights into how ripening affects both the chemical composition and antioxidant properties of damson plums.

### 3.1 PH, dry matter, and stone weight

Throughout the ripening process, the pH of fruit typically undergoes changes, often attributed to shifts in chemical components, particularly the content of phenolic acids. Table 1 presents the pH values of various samples of damson plums as measured in the current experiment.

**TABLE 1:** Basic information about damson plum samples harvested at various stages of ripening.

Name	Early ripening	Mid ripening	Late ripening
pH	2.85 ± 0.07^a^	2.95 ± 0.07^ab^	3.00 ± 0.01^b^
Dry matter (%)	21.03 ± 1.54^a^	19.73 ± 1.02^a^	19.33 ± 1.05^b^
Stone weight (%)	30.23 ± 1.16^a^	29.67 ± 2.93^a^	24.30 ± 0.80^b^

*Note*: The values with different superscripted letters within the same row indicate significant (*p* < .05) differences.

Notably, this data reveals a consistent upward trend in pH corresponding to the ripening stages; specifically, the pH of damson plums exhibited a significant (*p* < .05) increase from the early‐ripening stage (2.85 ± 0.07) to the late‐ripening stage (3.00 ± 0.01). These findings strongly indicate a reduction in acidic substances during the ripening process, potentially contributing to the sweeter taste of plums. This aligns with previous research (Vlaic et al., 2014), where an analogous increase in pH (from 3.14 to 3.74) was observed in plum fruit during different harvesting phases. Furthermore, the accumulation of sugars in the fruit as ripening progresses (Gil et al., 2002) offers a plausible explanation for the pH elevation observed in our study as a consequence of the harvesting time.

The dry matter content of the plum samples in this experiment decreased from 21.03% to 19.33% during the ripening stages (Table 1). The reported values for dry matter content for the Elena variety (*Prunus domestica*) (19.1%) (Walkowiak‐Tomczak et al., 2008) are similar to the dry matter content of damson plums (19.33%) found in the late‐ripening stage in the present study. A similar decreasing trend (28.3%–25%) with the ripening stage (1st day to 8th day) has also been found for other stone fruits such as avocado (Ozdemir & Topuz, 2004).

The water‐holding capacity in this experiment increased with the progression of the ripening stages (confirmed by moisture analysis in Table 2), which explains the decreasing trend of dry matter content as the result of the fruit ripening. Similar to dry matter, the stone weight of plums (stone:flesh ratio) decreased with the progression of the harvesting phases (Table 1). The stone weight of damson plums dramatically decreased from around 30.23% at the early‐ripening stage to around 24.30% at the late‐ripening stage. Thus, late‐ripening fruit contained higher water and nutrient contents than the fruit harvested at the early‐ripening stage, which could increase the flesh weight of the ripened fruit. The stone might attain a fixed weight at the early‐ripening stage (Del Rio & Caballero, 2008), and only the edible portion gains further weight throughout the ripening process, due to the accumulation of nutrients and bioactive compounds. This might be the reason for the low stone weight (%) in late‐ripening samples compared to the fruits harvested in the early‐ or mid‐ripening stages.

**TABLE 2:** General nutrient information about fresh damson plum samples harvested at different stages of ripening.

Name	Early ripening	Mid ripening	Late ripening
Moisture (%)	81.70 ± 1.27^b^	82.70 ± 0.86^ab^	83.13 ± 0.80^a^
Ash (%)	0.60 ± 0.07^a^	0.57 ± 0.04^a^	0.57 ± 0.04^a^
Fat (%)	1.07 ± 0.18^a^	0.83 ± 0.15^ab^	0.77 ± 0.04^b^
Protein (%)	1.00 ± 0.12^a^	0.83 ± 0.08^a^	0.90 ± 0.12^a^
Carbohydrate (%)	11.45 ± 0.21^a^	11.60 ± 0.14^a^	11.65 ± 0.35^a^
IDF (%)	1.75 ± 0.21^a^	1.55 ± 0.21^a^	1.45 ± 0.21^b^
SDF (%)	1.40 ± 0.14^a^	1.20 ± 0.14^b^	1.05 ± 0.21^c^
TDF (%)	3.15 ± 0.35^a^	2.75 ± 0.35^ab^	2.50 ± 0.42^b^
Energy (kJ/100 g)	280.3 ± 5.94^a^	267.1 ± 8.63^b^	263.6 ± 14.21^b^

*Note*: % refers to g/100 fresh samples. The values (mean ± *SD*) in a row with different superscript letters differ significantly (*p* < .05).

Abbreviations: IDF, insoluble dietary fiber; SDF, soluble dietary fiber; TDF, total dietary fiber.

### 3.2 Proximate composition and other nutrient analysis

The proximate compositions, including moisture, ash, fat, protein, carbohydrates, insoluble dietary fiber (IDF), soluble dietary fiber (SDF), total dietary fiber (TDF), and energy, are shown in Table 2. All fruit samples contained high moisture content (Table 2), which is consistent with the moisture content reported for other types of plums (86%–88%), or other stone fruits such as apricot (85%–86%), cherry (77%–84%), peach (87%–90%), and nectarine (86%–88%) (Wills et al., 1983). Similar to damson plums, European plums (*Prunus domestica*) and plums from the Japanese variety (*Prunus salicina*) also contain high moisture content (Igwe & Charlton, 2016). In our study, the late‐ripening fruit was found to contain a higher moisture content compared to fruit in the early‐ripening stage (83.13% vs 81.70%, respectively). Ash, protein, and carbohydrate contents were not affected by the ripening stages (Table 2).

However, the results showed a decreasing trend for dietary fiber content, including IDF, SDF, and TDF, from the early‐ripening stage to the late‐ripening stage (Table 2). Due to the reductions of fat and carbohydrates as a result of ripening stages, the energy decreases from the early‐ripening stage (280.30 ± 5.94 kJ/100 g) to the late‐ripening stage (263.55 ± 14.21 kJ/100 g). The decreasing effect of energy has also been observed for banana (from 8.8 MJ/kg to 8.1 MJ) and plantain (9.7 to 8.9 MJ/kg) samples with the progression of the ripening stages, which is consistent with our study (Emaga et al., 2011). Adenosine triphosphate (ATP) and adenosine diphosphate (ADP content) are reported to decrease with the progression of ripening stages of peach fruit samples (Jin et al., 2013), which might explain the reason for the decreasing trend of energy content in our samples as the fruit matured.

### 3.3 Sugar profile

Seven types of sugar analyses were carried out to investigate the sugar profile of New Zealand damson plums. The sugar information of these plums at different stages of ripening is shown in Table 3.

**TABLE 3:** Sugar profiles of fresh damson plum samples harvested at different stages of ripening.

Sugar content (% of flesh mass)	Early ripening	Mid ripening	Late ripening
Fructose (%)	1.85 ± 0.21^a^	1.80 ± 0.00^a^	1.90 ± 0.14^a^
Glucose (%)	4.05 ± 0.35^a^	4.10 ± 0.14^a^	4.35 ± 0.49^a^
Lactose anhydrous (%)	<0.1	<0.1	<0.1
Lactose monohydrate (%)	<0.1	<0.1	<0.1
Maltose (%)	<0.1	<0.1	<0.1
Sucrose (%)	0.90 ± 0.14^b^	1.10 ± 0.00^a^	0.75 ± 0.07^b^
Galactose (%)	<0.1	<0.1	<0.1
Total sugar (%)	6.80 ± 0.42^a^	7.00 ± 0.14^a^	7.00 ± 0.57^a^

*Note*: The values (mean ± *SD*) in a row with different superscript letters differ significantly (*p* < .05).

Three of the sugars, including fructose, glucose, and sucrose, were detected and correspondingly quantified in the damson plum samples, while the others (i.e., lactose, maltose, and galactose) were found to be present under the detection limit (Table 3). Lactose is milk sugar, and its presence in plum samples was not expected anyway. Fructose and glucose were found to be the two major sugars in the fresh plums. Glucose (4.0%–4.35%) and fructose (1.8%–1.9%) contents in damson plums in this study were quite similar to the glucose (4.0%–4.6%) and fructose (0.82%–1.8%) contents of green plums reported by Saridaş et al. (2016). However, damson plums in this experiment contained lower levels of glucose, fructose, and sucrose compared to the contents of glucose (5%), fructose (3.07%), and sucrose (1.57%) reported for European plums (Mohapatra et al., 2016).

The content of both fructose and glucose remained stable during the ripening process (Table 3). The other observation was that the concentration of glucose was two times higher than that of fructose. The content of sucrose increased significantly from 0.90% at the early‐ripening stage to 1.10% at the mid‐ripening stage and then dropped significantly to about 0.75% at the late‐ripening stage. The sucrose content of damson plums in the current study was higher than the reported sucrose content for green plums (0.06%–0.14%) reported previously (Saridaş et al., 2016). Garcia‐Marino et al. (2008) investigated the soluble sugars in edible and nonedible parts of damson plums (fruits) during their development and ripening. Consistent with our results, they found that sucrose, glucose, and fructose were the three major sugars in these fruits during ripening. Interestingly, these authors (Garcia‐Marino et al., 2008) reported that the ratio of sucrose:glucose:fructose in the ripened whole fruit was 5:3:1. Sucrose was the most abundant (77 ± 8 mg/g fresh weight) soluble sugar in that study. However, in the case of New Zealand‐grown damson plums evaluated in the present study, we found that sucrose was the least abundant (1.1 ± 0.01 mg/g fresh weight peak value) sugar among the three soluble sugars, with a glucose:fructose ratio of 2.2:1 throughout the ripening stages. Nevertheless, the total sugar content did not vary significantly (*p <* .05) during the entire ripening period.

### 3.4 Vitamins

In the current investigation, vitamins A, C, D3, and E were detected and quantified in the damson plums at different stages of ripening (Table 4).

**TABLE 4:** Vitamins contents of fresh damson plum samples harvested at different stages of ripening.

Name	Early ripening	Mid ripening	Late ripening
Vit C (μg/100 g)	770 ± 0.50^c^	900 ± 0.80^b^	970 ± 17^a^
Vit E (μg/100 g)	850 ± 0.70^b^	850 ± 0.70^b^	900 ± 14^a^
Vit D3 (IU/100 g)	1.67 ± 0.09^a^	1.60 ± 0.08^a^	1.53 ± 0.09^b^
Vit A (μg/100 g)	4.20 ± 0.29^a^	3.93 ± 0.21^b^	3.87 ± 0.21^c^

*Note*: The values (mean ± *SD*) in a row with different superscript letters differ significantly (*p* < .05).

Vitamins A and D3 showed a decreasing trend with the progression of ripening stages. All plum samples were found to contain a considerable amount of vitamins C and E, both of which are reported to exhibit a strong antioxidative effect (Choi et al., 2004; Garg & Bansal, 2000; Suhail et al., 2012). The concentrations of vitamins C and E exhibited an increasing trend with the process of maturation in damson plums. With the progression of the ripening period, the plums accumulate higher moisture content (Table 2), which could retain more vitamin C due to their water‐soluble nature. However, the vitamin C content could decrease as an effect of processing. For instance, the drying method allows for moisture removal, which, in turn, concentrates sugars and other nutrients, resulting in a decrease in vitamin C content (Kaya et al., 2010). Another study (Yousef et al., 2016), which determined the effect of ripening stages on ascorbic acid content in Japanese plums, has also found an increasing trend of this vitamin over the ripening time (from 22.83 to 64.20 mg/100 g fresh weight).

### 3.5 Minerals

Seven minerals, including calcium, magnesium, potassium, sodium, phosphorus, selenium, and zinc, were detected and quantified in the present study (Table 5). Potassium was found to have the highest content of minerals in the damson plum samples, followed by calcium, phosphorus, magnesium, sodium, and zinc. Potassium is considered one of the major shortfall nutrients in the Western diet, such as the American diet (Spahn et al., 2011). In this study, there was no significant difference (*p* > .05) in the concentrations of potassium and sodium among the three ripening stages (Table 5). This study shows that damson plums are a rich source of dietary potassium, which, based on the evidence from the literature, could improve cardiovascular and bone health, resulting in a reduction in the risk of stroke and coronary heart disease (Weaver, 2013).

**TABLE 5:** Minerals information of fresh damson plum samples harvested at various stages of ripening.

Mineral content (mg/kg)	Early ripening	Mid ripening	Late ripening
Calcium	243.5 ± 6.36^a^	214.5 ± 2.12^b^	206 ± 12.73^b^
Magnesium	125.0 ± 4.24^a^	112.5 ± 0.71^b^	112 ± 8.49^b^
Potassium	3082.5 ± 244^a^	2853.5 ± 31.82^a^	2860.5 ± 273.65^a^
Sodium	10.00 ± 0.42^a^	9.6 ± 0.42^a^	9.45 ± 0.64^a^
Phosphorus	213.67 ± 5.19^a^	190.0 ± 16.06^b^	203.7 ± 5.44^ab^
Selenium	<0.02^a^	<0.02^a^	<0.02^a^
Zinc	1.05 ± 0.21^a^	0.95 ± 0.21^a^	0.95 ± 0.21^a^

*Note*: The values (mean ± *SD*) in a row with different superscript letters differ significantly (*p* < .05).

The potassium content of damson plums measured in this study (286–308 mg/100 g wet fruit) is higher than the values reported for other plum varieties (120–170 mg/100 g), peach (180–220 mg/100 g), cherry (200–280 mg/100 g), and nectarine (220–240 mg/100 g) (Wills et al., 1983). However, apricot is reported to contain higher potassium (320–350 mg/100 g) compared to damson plum (Wills et al., 1983). The sodium content of damson plums is within the range of the sodium content reported for other plum fruits (Wills et al., 1983). Damson plums contain higher potassium content than lower sodium content, which is recommended for the prevention of cardiovascular disease (Noubiap et al., 2015).

Regardless of the ripening stages, all plum samples in the current investigation contained a considerable amount of calcium and phosphorus, which could be beneficial for bone health. A previously published study (Rendina et al., 2012) investigated the effects of dried plum on the changes in bone metabolism and the immune response associated with ovarian hormone deficiency in rats and found that a specific compromise in trabecular bone in rats was prevented by the higher doses of dried plum (Rendina et al., 2012).

The results of the current study showed a decreasing trend in calcium, magnesium, and phosphorus contents with the progression of plums' ripening stages (Table 5). In particular, the calcium content significantly (*p* > .05) decreased from around 243.50 mg/kg in the early stage of ripening to around 206.00 mg/kg in the late stage. Calcium plays an important role in elongation, cell division, fruit growth, and the ripening process (Serrano et al., 2004). Similarly, magnesium remarkably decreased from 125 mg/kg (the early‐ripening stage) to 112 mg/kg (the late‐ripening stage). Similar to our study, long ago, another study (Taylor et al., 1993) reported that the Songold plums harvested at the late stage of the ripening period contained lower Ca and Mg contents compared to the same plums harvested at the early‐ripening stage. The fruits usually become softer with the reduction of calcium content (Serrano et al., 2004), which might explain the reason for the lower calcium content in the plums harvested at the late‐ripening stage in the current investigation, compared to those harvested at the early‐ripening stage. Mg is reported to improve plant growth through increasing Fe activity in the chloroplast and is responsible for photosynthesis and fruit color development (Serrano et al., 2004). The late‐ripening fruit might utilize more Mg for color development (Adeyemi & Oladiji, 2009), which might be a reason for the lower Mg content in the late‐ripening samples compared to the Mg content of the early‐ripening samples in the current experiment. There were no significant differences in the zinc content of damson plums among the three ripening stages. Other stone fruits, including other types of plum, apricot, cherry, nectarine, and peach, contain approximately 1 mg zinc per kilogram of edible portion (Wills et al., 1983), which is consistent with our findings in this study. The lower zinc content in plum samples also indicates that such a fruit has been grown in an unpolluted area and is completely safe for human consumption (Osmanović et al., 2014).

### 3.6 Phenolic content and antioxidant activity

**FIGURE 2:**
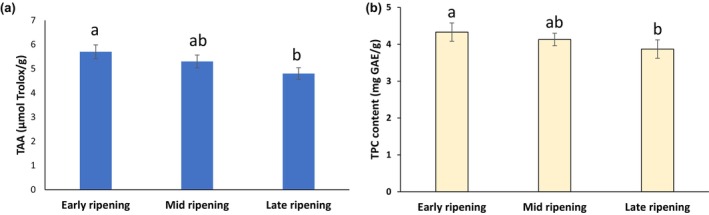
Total antioxidant activity (a) and total phenolic content (b) of fresh damson plums at various stages of ripening. The values (mean ± *SD*) in a row with different superscripted letters differ significantly (*p* < .05). GAE, gallic acid equivalent; TAA, total antioxidant activity; TPC, total phenolic content.

Plums are reported to contain considerable concentrations of antioxidants, mainly from phenolic compounds and vitamins (e.g., C and E). Figure 2 presents the total phenolic content (TPC) and total antioxidant activity (TAA) of the damson plums in this experiment, which were determined at various stages of ripening. TPC values showed a significant (*p* > .05) decrease by about 4.33 mg GAE/g to about 3.87 mg GAE/g as the maturity of damson plums progressed (Figure 2).

Another study (Mousavi et al., 2019) determined the TPC of damson plums in four granulometric classes, including 180, 180–315, 315–500, and >500 μm, and reported that the samples with different granulometric sizes showed different phenolic properties. Plum powder (>500 μm) contained the highest TPC (25.6 mg GAE/g dry weight (DW)), and the other granulometric sizes of damson plums contained TPC values ranging between 21.1 and 18.8 mg GAE/g DW, which are consistent with the TPC values we obtained for the New Zealand‐grown damson plums in this report; i.e., 23.7, 23.9, and 22.9 mg GAE/g DW in the samples harvested during the early‐, mid‐, and late‐ripening stages, respectively. These results are interesting, as the TPC content in these plum samples is higher than some antioxidant‐rich fruits such as mango (2.4 mg GAE/g), tamarind (3.9 mg GAE/g), longan (1.6 mg GAE/g), avocado (1.3 mg GAE/g), jackfruit (0.9 mg GAE/g), blackberries (2.5 mg GAE/g), cranberries (3.15 mg GAE/g), raspberries (1.26 mg GAE/g), and strawberries (2.25 mg GAE/g) (Fu et al., 2011; Soong & Barlow, 2004). Even some coffee samples, including ground Arabica coffee (3.6 mg GAE/g) and green Robusta (2.6 mg GAE/g) coffee samples (Olechno et al., 2020), contain less TPC than the damson plums tested in the current study. This indicates that the damson plums grown in New Zealand can be used as a functional antioxidant‐rich food ingredient that may be incorporated into various food formulations, with potential health‐promoting properties related to antioxidants.

Plums, in general, have been shown to have a high content of polyphenols (de Pascual‐Teresa et al., 2000; Karaman et al., 2013). Polyphenols, such as phenolic acids and flavonoids, are one of the most important groups of chemical compounds in these stone fruits. These compounds have a high free radical scavenging ability, which can play an important role as effective natural antioxidants in our daily diet (Pokorný, 2007). Polyphenols are reported to contain health‐promoting properties such as inhibiting the growth of human prostate cancer cells (Chun, Kim, Moon, et al., 2003; Kampa et al., 2000). Moreover, polyphenols, especially catechins (present in damson plums), may also induce the apoptosis of endothelial cell proliferation (Sartippour et al., 2001) and suppresses the growth of breast cancer cells (Damianaki et al., 2000).

The present study used ABTS scavenging capacity to evaluate the TAA of the New Zealand damson plums. The data presented in Figure 2 indicated that the New Zealand‐grown damson plums had a notable ability to scavenge ABTS free radicals. In line with the results of previously published research (Mubarak et al., 2012), the findings of this study agreed that the phytochemicals from damson plums may supply substantial natural antioxidants to provide antioxidant activity effects. The current data also showed that the content of polyphenols could decrease with the progression of the maturity of the fruit (Figure 2). As the plums were harvested at different ripening stages, their antioxidant capacities and phytochemical content would be different. These results correspond to the observation of decreasing polyphenol content in the brown apple as the result of the ripening stages (Mokhtar et al., 2021; Murata et al., 1995). Another study (Mokhtar et al., 2021) also found a higher amount of polyphenols in the early stage of maturity (77.5 mg GAE/100 g wet weight) of pumpkins compared to ripe pumpkins (55.60 mg GAE/100 g wet weight). The TPC might decrease due to the oxidation of polyphenols caused by the enzyme known as polyphenol‐oxidase (Fawole & Opara, 2013; Kulkarni & Aradhya, 2005). This might explain the reason for the decrease in polyphenol content in damson plum samples (Figure 2) with the progression of ripening stages. Based on the literature (Chun, Kim, & Lee, 2003), the major polyphenols in damson plums are chlorogenic acids, followed by cyanidin glycosides. The composition of these two polyphenolics would affect the TAA of plum cultivars. For better identification of the phenolic composition of the New Zealand damson plums, further research is currently undergoing in our laboratories to separate and quantify various fractions of polyphenols from these plums using chromatographic techniques such as high‐pressure liquid chromatography with mass spectroscopy (HPLC/MS–MS).

## 4 CONCLUSIONS

Taken together, this study showed that the damson plums grown in New Zealand are a rich source of various macro‐ and micronutrients, as well as phenolic/antioxidant compounds, meaning that they could be used as a functional ingredient in the food and nutracutical industries. As the harvesting time/ripening stages progressed, there was a significant effect (*p* < .05) seen on the concentration of most nutrients, as well as phenolic and antioxidant properties of the New Zealand damson plums. Therefore, this data suggests that it is important to choose the right time for the harvest of stone fruits such as plums, which will ensure a healthy nutritional profile and maximum health benefits. Based on the data from the current experiment, the suitable time for the harvest of damson plums in New Zealand appears to be when the fruit is at its early–mid ripening stage. This is the very first comprehensive research on the nutritional profile of damson plums and the effect of ripening, which will open the door for future research in this area. Further research is currently undergoing in our laboratories for the extraction, identification, isolation, and quantification of the individual phenolic compounds that exist in the New Zealand damson plums. A freeze‐dried damson plum powder that has been manufactured in the current study can be used as a functional food ingredient for various food formulations. This information will be useful for the plum industry, as well as the food manufacturers who may use this type of fruit as a natural ingredient for the manufacture of various food products.

## AUTHOR CONTRIBUTIONS

**Ali Rashidinejad:** Conceptualization (lead); data curation (equal); formal analysis (equal); funding acquisition (lead); investigation (equal); methodology (lead); project administration (lead); resources (lead); software (equal); supervision (lead); validation (lead); visualization (equal); writing – original draft (equal); writing – review and editing (lead). **Mirja Kaizer Ahmmed:** Data curation (equal); formal analysis (equal); software (equal); validation (equal); visualization (equal); writing – original draft (equal).

## CONFLICT OF INTEREST STATEMENT

There are no conflicts of interest in this manuscript.

## ETHICS STATEMENT

Ethics approval was not required for this research.

## Data Availability

All corresponding data are presented within this study.
